# Grassland Arthropods Are Controlled by Direct and Indirect Interactions with Cattle but Are Largely Unaffected by Plant Provenance

**DOI:** 10.1371/journal.pone.0129823

**Published:** 2015-07-09

**Authors:** Kelly Anne Farrell, W. Stanley Harpole, Claudia Stein, Katharine N. Suding, Elizabeth T. Borer

**Affiliations:** 1 Department of Zoology, Oregon State University, Corvallis, OR, United States of America; 2 Department of Ecology, Evolution and Organismal Biology, Iowa State University, Ames, IA, United States of America; 3 Department of Biology, Washington University in St. Louis, St. Louis, MO, United States of America; 4 Environmental Science, Policy & Management, University of California at Berkeley, Berkeley, CA, United States of America; 5 Department of Ecology, Evolution, and Behavior, University of Minnesota, St. Paul, MN, United States of America; Institute of Plant Physiology and Ecology, CHINA

## Abstract

Cattle grazing and invasion by non-native plant species are globally-ubiquitous changes occurring to plant communities that are likely to reverberate through whole food webs. We used a manipulative field experiment to quantify how arthropod community structure differed in native and non-native California grassland communities in the presence and absence of grazing. The arthropod community was strongly affected by cattle grazing: the biovolume of herbivorous arthropods was 79% higher in grazed than ungrazed plots, whereas the biovolume of predatory arthropods was 13% higher in ungrazed plots. In plots where non-native grasses were grazed, arthropod biovolume increased, possibly in response to increased plant productivity or increased nutritional quality of rapidly-growing annual plants. Grazing may thus affect plant biomass both through the direct removal of biomass, and through arthropod-mediated impacts. We also expected the arthropod community to differ between native and non-native plant communities; surprisingly, arthropod richness and diversity did not vary consistently between these grass community types, although arthropod abundance was slightly higher in plots with native and ungrazed grasses. These results suggest that whereas cattle grazing affects the arthropod community via direct and indirect pathways, arthropod community changes commonly associated with non-native plant invasions may not be due to the identity or dominance of the invasive species in those systems, but to accompanying changes in plant traits or functional group composition, not seen in this experiment because of the similarity of the plant communities.

## Introduction

Through the intentional and incidental introduction of non-native plant species into grasslands, coupled with heavy agriculture and livestock grazing, many of the world’s grasslands have experienced significant changes in plant composition and diversity [1,2]. Although the direct effects of these changes on plant composition have received substantial attention, less is known about the effects on consumers in these altered ecosystems. Understanding the effects of grazing and grassland invasion on arthropods represents a critical gap because of the key role arthropods play in the composition and function of grasslands. Arthropods can control the net primary production of grasslands [3,4], can control nutrient cycling and decomposition rates [4–8], can serve as plant pollinators [9,10], and can vector diseases [11]. Arthropod diversity often covaries with plant diversity [7,8], plant functional group diversity [8], plant community composition [12], or plant productivity [13,14]. Because of this relationship between arthropod and plant communities, changes in plant community due to grazing or non-native plants may precipitate altered arthropod community composition and hence to altered ecosystem function. We currently have little understanding of the dominant mechanisms and outcomes of these perturbations or whether their effects may synergistically impact grassland arthropods.

The introduction of cattle to natural grasslands can result in region-wide changes that could impact arthropod communities. Plant biomass, which decreases with cattle grazing, is positively associated with arthropod abundance [7], and taller grasses host more insect species than short grasses [15]. Arthropod diversity and abundance increase with the structural complexity of vegetation [16,17] with greater predator abundance and diversity associated with the complex vegetation structure of ungrazed habitats [15,18]. Alternately, grazing may increase herbivore abundance by increasing plant productivity and the relative abundance of nutritious new growth [15,19]. Grazing may then have opposite effects on different groups of insects, causing an increase in groups dependent on the nutritional quality of the plants but a decrease in groups that require structural complexity.

Plant invasion represents another region-wide perturbation that can alter the overall nutritional quality, structural profile, or functional group makeup of a plant community. These alterations could change the resources available to phytophagous insects and their predators and parasitoids. In California, the focal region for our study, 9.2 million hectares of historically perennial-dominated grassland have been invaded by annual non-native grasses, their invasion likely facilitated by overgrazing and drought during the 19^th^ century [20]. Whereas in many habitats the invasion of non-native plants may decrease plant diversity [21,22] and lead to declines in arthropod diversity [23], the abundance of non-native plants is not necessarily an indicator for decreased plant species richness or changes in plant functional group composition. Instead, non-native grasses rarely cause significant reductions in native plant community richness in Mediterranean ecosystems such as those found in California [22]. However, the importance of plant composition to arthropods [12] suggests that this grassland conversion could significantly alter the region’s arthropod community composition by increasing the prevalence of annual grasses. Annual grasses tend to support lower herbivorous insect diversity than expected by chance [15], but can support substantially higher herbivorous insect fecundity [24]. We expect, therefore, that non-native annual grasses will support lower insect diversity and changed arthropod community compared to native perennial grasses.

Plant invasions in combination with changes in grazing may act synergistically or have feedback effects that reinforce an altered grassland flora via a variety of pathways. If exotic plants are more tolerant of cattle grazing and insect herbivory than native plants, and at the same time if exotic plants can sustain larger populations of herbivorous insects than native plants, cattle grazing may initiate a positive feedback process that leads to increased exotic plant dominance [25]. Alternately, grazing might indirectly reduce predator biomass by reducing plant complexity and cover; thus may release arthropod herbivores and increase the rate of invertebrate herbivory in plots grazed by cattle [26]. This indirect interaction chain would likely favor faster-growing annuals over perennial plants. Recent work suggests a third pathway, including disease. In this case, vertebrate herbivores may increase the proportion of highly-competent non-native hosts of barley yellow dwarf virus [27], leading to increased arthropod vector reproduction [24], which may sustain domination of the landscape by non-native grasses [28]. Overall, an alternate, annual grass dominated state maintained by any of these interacting processes would likely support a less diverse arthropod herbivore assemblage, dominated by a few species [18]. Here we experimentally examined arthropod richness, evenness, biomass, and compositional responses to the effects of cattle grazing and invasive plant species and their interaction. We used an experimental manipulation of grass species composition (native perennial mixture versus non-native annual mixture) crossed with the effects of a cattle grazing treatment to test: (1) how cattle grazing affects arthropod communities and (2) how the arthropod community differs in an invaded plant community compared to a native plant community. Specifically, we predict that the proportion of predatory arthropods should decrease with cattle grazing because of the reduction in available plant biomass that can support herbivorous arthropods. Finally we ask (3) whether the effect of grazing on arthropod communities is dependent on the composition of the plant community (native or non-native).

## Methods

### Experimental design

**Fig 1:**
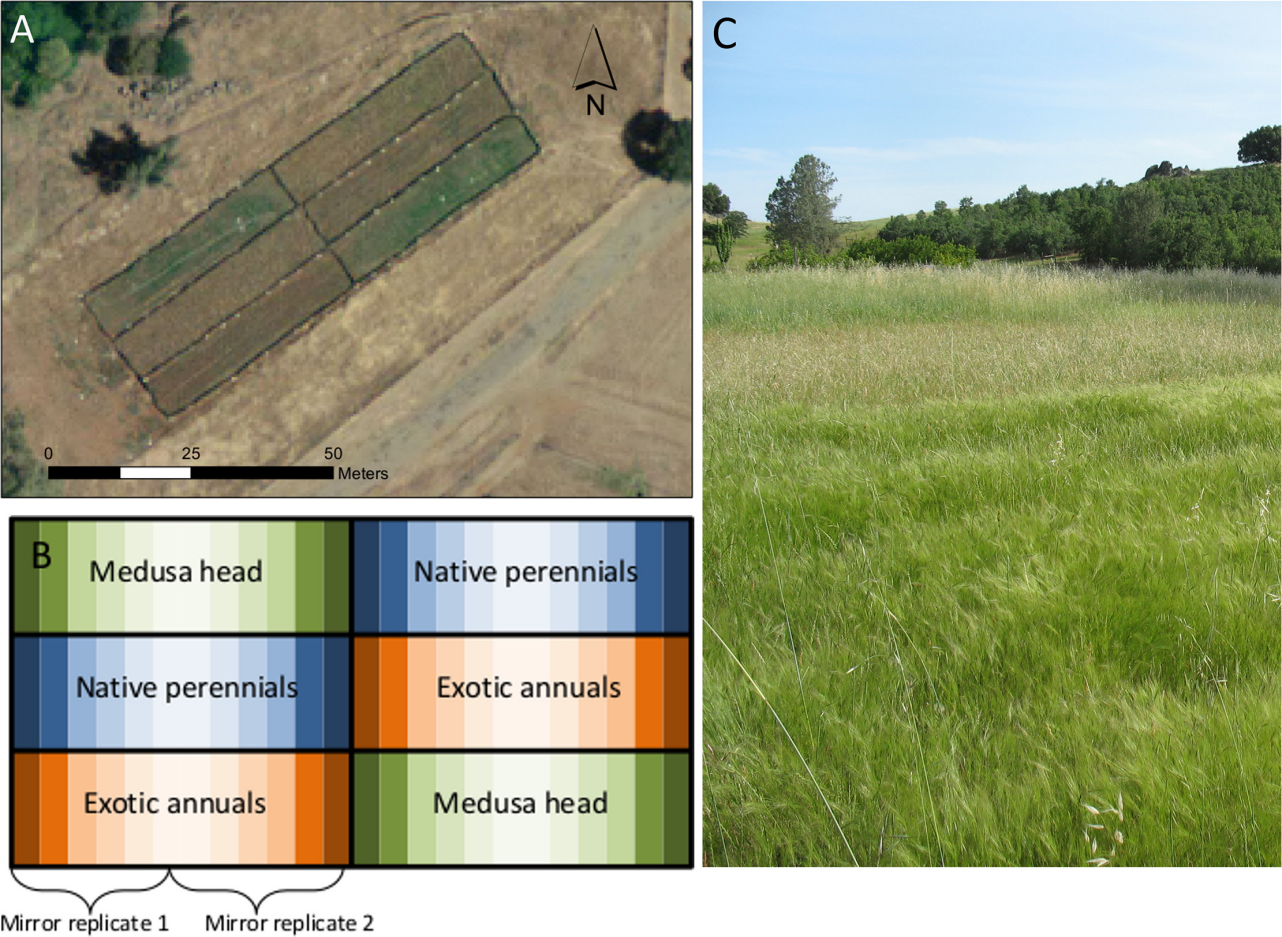
Plot experimental treatments. (A) A satellite photo of one of the experimental fields in 2010, courtesy of the U.S. Geological Survey. (B) A diagram of the experimental setup of one of the fields. Plots were solarized in 2006, then planted with an exotic annual grass mixture, a native perennial grass mixture, an exotic Medusae head (*Taeneathrum caput-medusae*) grass monoculture, or left as a control in late 2006 and early 2007. Across these plantings, cattle herding and mowing were combined to create a gradient of grazing intensity, from heavy grazing at the centers of the plots (lightest color) to no grazing at the outside of the plots (darkest color). Two mirror replicates of each grass * grazing block were sampled in this study, for a total of four samples from each grass*grazing combination in each field. (C) The vegetation differences between grass treatments were highly evident at the arthropod sampling date in May 2009.

To study the effects of cattle grazing and plant composition on grassland arthropods, we studied arthropod communities in a randomized factorial experiment at the University of California Sierra Foothills Research and Extension Center (SFREC) in Browns Valley, California, USA (39° 15' N, 121° 17' W). The experiment was replicated in two pastures that were tilled and solarized in October 2006 to remove vegetation and decrease soil seed bank and pathogens. We sampled arthropods from two grass provenance treatments within this experiment: (1) native perennial bunchgrasses planted as plugs (*Stipa pulchra*, *Elymus glaucus*, *Melica californica)* and (2) non-native annual grasses established by seeding (*Avena fatua*, *Bromus hordeaceus*, *Festuca perenne*). These were replicated with two blocks of each provenance treatment established on each pasture for a total of 4 blocks. Two years after establishment, a gradient of 6 grazing treatment levels was initiated, consisting of both trampling by livestock and mechanical mowing. The most intensive grazing + mowing treatment, leaving 220–330 kg of residual dry matter per ha, was based on estimates from intensively grazed rangeland bordering this research site. For logistical reasons, the highest grazing intensity was in the centermost plots, with decreased intensity moving outwards in both directions. This resulted in two mirror replicates within each grass treatment block, for a total of eight 3m × 10m plots of each grazing × grass factorial manipulation (Fig 1).

The intensive grazing + mowing treatment (“heavily grazed”) plots were trampled in late March 2008 and 2009 when plants started flowering and in June 2008 when most plants were senescent. 40–42 cattle (up to one year old, Black Angus Mix, approximately 400–450 kg/head) were herded for 30–45 minutes during each trampling treatment. These cattle were herded as part of the normal research and extension activities at SFREC under the facility’s protocol for animal use and care. In addition to cattle trampling, plots were mowed to a height of 2 cm above the ground, and biomass removed, three times during 2008 (before each trampling and in late February during early plant growth) and twice during 2009. This combination of trampling and mowing simulated, as closely as possible, the impacts of grazing within the spatial scale of the treatment plots. No permits were required for the described study, which complied with all relevant regulations and was approved by the research advisory board of SFREC. No endangered or protected species were involved in this study.

### Vegetation sampling

Plant species abundances, aboveground biomass, and litter mass were sampled during peak biomass in May 2009 from areas in each plot adjacent to arthropod sampling quadrats. Percent cover of each vascular plant species within a 1m^2^ quadrat was visually estimated. Aboveground plant biomass was sampled in a 0.25 × 0.25 m square by cutting the vegetation 2 cm above ground. Plant litter was collected from the same 0.25 × 0.25 m square. Residual dry matter (RDM), the plant material left on the ground following a growing season and which is a standard method to quantify grazing intensity in rangeland management [29], was measured just before the start of a new growing season in September 2009. RDM was harvested in a 0.25 × 0.25 m square within each treatment combination. Aboveground biomass, litter, and RDM were dried at 60°C for 72 h and weighed.

### Arthropod sampling

We sampled arthropods from the ungrazed and most heavily grazed plots in the native perennial and non-native annual grass treatments in May 2009. Arthropods were vacuum sampled using a method similar to that of Stewart & Wright [30]. A fine mesh bag, fitted into the suction tube of a gas-powered leaf blower, was used to “comb” vegetation from the bottom up within a 1 m^2^ quadrat for 30 seconds. Bagged insects were stored on ice and frozen upon return to the laboratory. Two samples were taken from each plot and combined during analysis to overcome within-plot spatial variability in arthropod distribution. While this method of suctioning does not thoroughly sample large and mobile arthropods which can escape the open quadrat, it is effective for sampling many groups of small, vegetation-inhabiting arthropods. Importantly, it provides samples of the arthropod community that are unbiased with respect to the experimental treatments.

In the lab, all arthropods visible at 10x magnification were identified to morphospecies using a dissecting microscope to examine externally visible characters at up to 50x magnification. The first instance of each morphospecies was considered the primary voucher, photographed, and pinned. Subsequent individuals were identified using a digital library of photographed vouchers. Using taxonomic keys and expert advice, morphospecies were classified to the lowest possible taxonomic rank; the majority of morphospecies were identified to family (S1 Table). Trophic status (herbivore, carnivore, parasitoid, detritivore) was assigned to each morphospecies, based on information in published keys and family accounts (S1 Table). Where ambiguity in the trophic classification for a morphospecies was possible, it was considered “varied” and omitted from the trophic analysis. For instance, if the lowest taxonomic identifier (e.g. family) contained both herbivores and carnivores within California, no trophic group was assigned for that morphospecies. With the exception of parasitoids and specimens collected as immatures, adult food source was used if known.

Arthropod biovolume, an estimate of body size and of the amount of secondary production supported by the plant community, was estimated for all morphospecies. We use this measure as a surrogate for individual biomass. Up to three specimens of each morphospecies in each vacuumed sample were measured for length and width (2*r*) to the nearest 0.1mm, and biovolume was calculated as π*r* ^2^×length, such that body volume was estimated to be cylindrical. For analyses, the biovolume assigned to each morphospecies was estimated as the average of all measured individuals (S1 Table). Plant data and arthropod counts and metadata are available in S1 Dataset.

### Analysis

Species richness and Shannon evenness (H’ = H/ln(S) where S represents the number of species and H represents the Shannon-Weiner diversity index [31]) were calculated for both plants and arthropods in each plot, along with abundance and total biovolume of arthropods. Generalized linear models (glm) with a Poisson distribution were used to test the response of arthropod richness and abundance, predicted by grazing and grass treatment interactions, blocked by experimental pasture, and with plant richness, plant evenness, plant biomass, and litter mass as covariates. Models with normal or natural log distribution were developed to examine the response of arthropod evenness and biovolume to the experimental treatments. The full and reduced models were compared for goodness of fit and best fitting models were selected using the Akaike Information Criteria (AIC). Differences in plant species richness, evenness, biomass, and litter mass were analyzed between grass treatments using t-tests and between grazing treatments using paired t-tests. These analyses were performed in R (Version 2.8.1, R Foundation for Statistical Computing), using the MASS (Venables and Ripley 2002), lattice (Sarkar 2008), and vegan (Oksanen et al. 2010) packages.

To analyze the impacts of grazing and grass composition on the arthropod community, we compared the abundance of each morphospecies in each plot. The data from one grazed annual plot was excluded from the analyses because of insufficient labeling in the field. As a balanced design was needed, one randomly chosen ungrazed annual plot was also omitted from the analysis. Beginning with a matrix of 30 plots and 252 arthropod morphospecies, data were transformed by excluding morphospecies that occurred in only one plot, resulting in a matrix of 30 plots and 179 morphospecies. This process decreases the noise generated by rare species while increasing the detection of patterns in community relationships [32]. We scaled the abundance of each morphospecies between 1 (its highest abundance in any plot) and 0 (not occurring in a plot). This transformation is appropriate for data sets in which the abundance of one group may differ strongly from the abundance of another group; it prevents super-abundant taxa from masking patterns of less abundant taxa ([32]). All multivariate analyses were conducted in PC-ORD (McCune and Medford 2010, version 6.243 beta).

Permutational-based multivariate analyses of variance (PerMANOVAs) were used to test for differences in arthropod morphospecies composition between treatments, using Sørensen distance measure after the methods of Anderson [33]. The experimental design prevented analysis of all variables at once. The impact of grazing was analyzed by conducting a perMANOVA comparing morphospecies composition in grazed and ungrazed paired plots, blocked by grass treatment plot. The impact of grass treatment was analyzed separately within grazed and ungrazed treatments and was blocked by experimental field. In ungrazed plots, a matrix of 16 plots × 138 relativized species abundances were analyzed to compare arthropod composition in annual and perennial plots. In grazed plots, an additional 2 plots were randomly discarded (because of missing sample) to create a balanced design, leading to a final matrix of 12 plots × 122 relativized species abundances. Results were not qualitatively different when all grazed plots were included and when the 4 plots were omitted from the analyses.

We used a nonmetric multi-dimensional scaling (NMS) ordination with Sørensen distance measure to visualize the ways that environmental variables influenced arthropod community. Ordinations plotted each plot’s location in 179-dimension morphospecies-space, each axis representing the proportional abundance of a single morphospecies in a plot. Using a random starting configuration, 250 runs with real data, and 250 runs with randomized data, these dimensions were collapsed into the smallest number of axes that adequately explained the data. Environmental characteristics of each plot (i.e. treatments and plant community characteristics) were overlain onto the final ordination to estimate the correlations between the environmental variables and ordination axes.

Because the multivariate analyses identified a significant shift in the arthropod community with grazing, we tested whether closely related arthropod morphospecies could be treated as independent units within the analysis or whether phylogenetic groups were responding similarly, thus driving the trend. Using the program Mesquite (Maddison & Maddison 2010, version 2.74 (build 550)), a tree was built to reflect the phylogeny of morphospecies. The correlation of each morphospecies to grazing (S1 Table) was mapped onto the tree. We compared the number of squared changed steps in the observed data with the predictions of a null hypothesis in which the correlation values were distributed randomly with respect to the phylogeny. This null hypothesis represents a phylogeny in which there is no historical, phylogenetically-driven response to grazing. Values to test the null hypothesis were generated by a permutation test, in which we randomly shuffled the correlation values among taxa 10,000 times.

## Results

### Characterization of treatments

Plant diversity, dominance, and productivity differed with grazing intensity and between native and non-native grass treatments (Table 1). Plant species richness did not differ among grazing or grass treatments (overall average richness 14.4 species, grazing paired t-test p = 0.088, grass t-test p = 0.121). Average plant species evenness was higher in grazed than ungrazed plots (ungrazed 0.561 ± 0.0455; grazed 0.676 ± 0.0229; paired t-test p = 0.019) and lower in non-native annual plots (0.544 ± 0.0457) than in native perennial plots (0.693 ± 0.0143, t-test p = 0.006). In grazed plots, plant litter was 46% lower (paired t-test p<0.01) and RDM 69% lower (paired t-test p<0.001) compared to ungrazed plots. Although the mean biomass in grazed plots was 29% lower than in ungrazed plots at the time of collection, this difference was marginally non-significant (paired t-test p = 0.07).

**Table 1:** Responses of the plant community to experimental treatments.

	Plant Community Response, 95% Confidence Intervals			
	Richness (# species)	Shannon Evenness	Biomass (g/m^2^)	Litter mass (g/m^2^)
^§^Native perennial vs. exotic annual	-5.1, 0.6	-0.250, -0.0489**	-123.88, 57.70	-103.36, 19.20
^‡^Grazed vs. ungrazed	-0.4, 5.2	0.0214, 0.2089 *	-147.94, 6.20 †	-128.10, -27.98 **

Plant community metrics were compared between native perennial and non-native annual grass-dominated communities using a t-test, and between grazed and ungrazed treatments using paired t-tests. Negative numbers indicate that annual grass or grazed plots have a greater response value (richness, evenness, etc.) than perennial grass or ungrazed plots. The 95% confidence interval range is shown for each test. (Significance levels: **, p<0.01; *, p< 0.05; †, p<0.1).

^§^Values in row represent the 95% confidence range for a difference in the plant community response between plant provenance/life history treatments {native perennial vs. non-native annual grass plots}.

^‡^Values in row represent the 95% confidence range for a difference in the plant community response between grazing treatments {grazed vs. ungrazed plots}.

### Arthropod community responses

**Fig 2:**
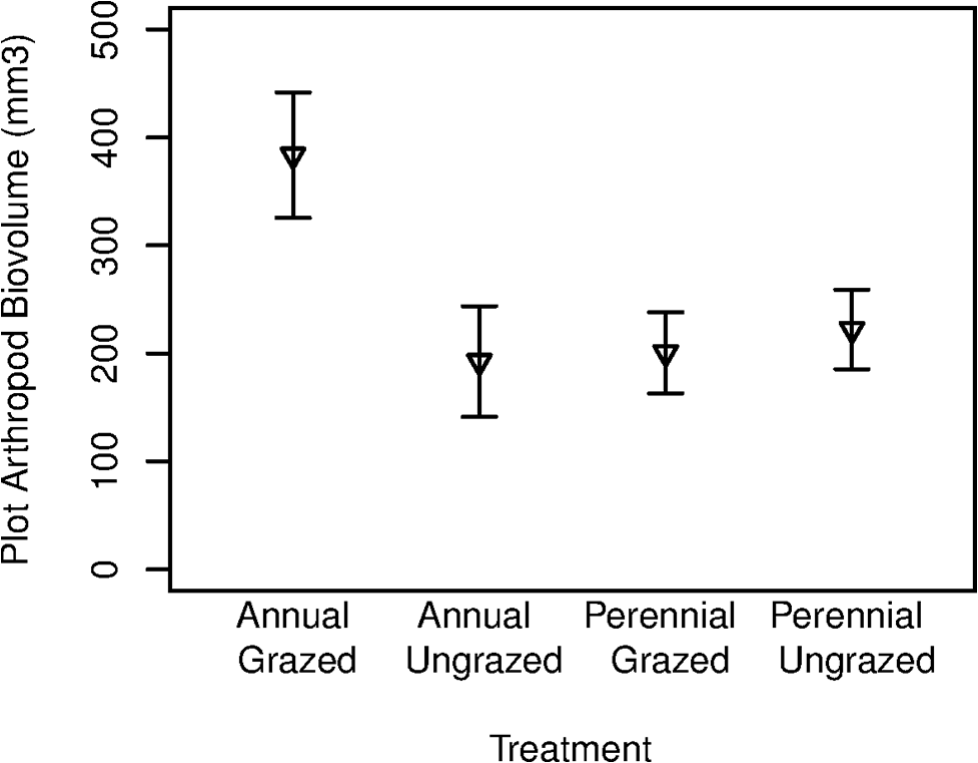
Response of total plot arthropod biovolume to grass provenance and grazing treatments. Values represent means ±SE.

Across all experimental plots, 27,927 arthropods representing 252 morphospecies were collected. We found 42 herbivorous, 29 predatory, 48 parasitoid, and 6 detritivorous arthropod morphospecies, and 53 morphospecies with trophic preferences that were unknown or too varied to classify. Average specimen length was 0.6mm and the largest specimen was 11.5mm long. Hemipterans, especially cicadellids and aphids, were found in high abundance, as were collembolans. The most diverse group was the microhymenopteran parasitoids (70 morphospecies; S1 Table). Neither grazing nor grass provenance treatments were correlated with changes in arthropod richness or evenness. Arthropod abundance was slightly lower with grazing and in non-native grasses compared to ungrazed or native grass plots. Abundance was positively correlated with the number of plant species in a plot (F-test p = 0.047) and arthropod species richness was positively correlated with the evenness of the plant community (F-test p = 0.009; Table 2). Total arthropod biovolume was higher in non-native grass treatments, but only in the presence of grazing, as indicated by a significant grazing × grass interaction (F-test interaction term p = 0.029 on 3, 27 df; Fig 2).

**Table 2:** Results of linear model testing for significant impacts of environmental parameters on arthropod diversity measures and biovolume of arthropod trophic guilds.

		Arthropod Diversity			Arthropod Biovolume				
Parameters		Abundance (log link)	Richness (log link)	Evenness	Total	Herbivore (ln herb)	Predator (ln pred)	Parasitoid	Detritivore
	Non-native grasses (compared to Native)	-0.166***	-	-	183.14*	0.4263	-0.8058	1.8439†	2.7246
	Grazing	-0.247***	-0.1567*	-	191.13**	0.8621*	-2.3813**	-	2.4928
	Grass × Grazing	-0.100**	-	-	212.77*	0.5889	-1.9326*	-	-
	Field/Block	-0.09***	-0.0222**	0.0005*	-	0.0917**	-0.1388*	-0.1771	-0.5525*
	Plant richness	0.035***	-	-0.0005*	-	-	-	-	-
	Plant evenness (Shannon)	0.503***	0.8877***	-	-	-	6.2731**	5.0651	14.1057*
	Plant biomass	0.0001*	-	0.00002	-	-	-	0.0063	-
	Litter mass	-0.002**	-0.0010	-	-	-	-0.0081†	-0.0109	-0.0243†

Environmental parameters included experimental treatments and plant community measures. Each model started with all parameters, and the best-fitting model was selected using AIC. For each arthropod response variable, the parameter estimates and their significance levels in the final model (***, p< 0.001; **, p<0.01; *, p< 0.05; †, p<0.1) are given. (ln) natural log.

**Fig 3:**
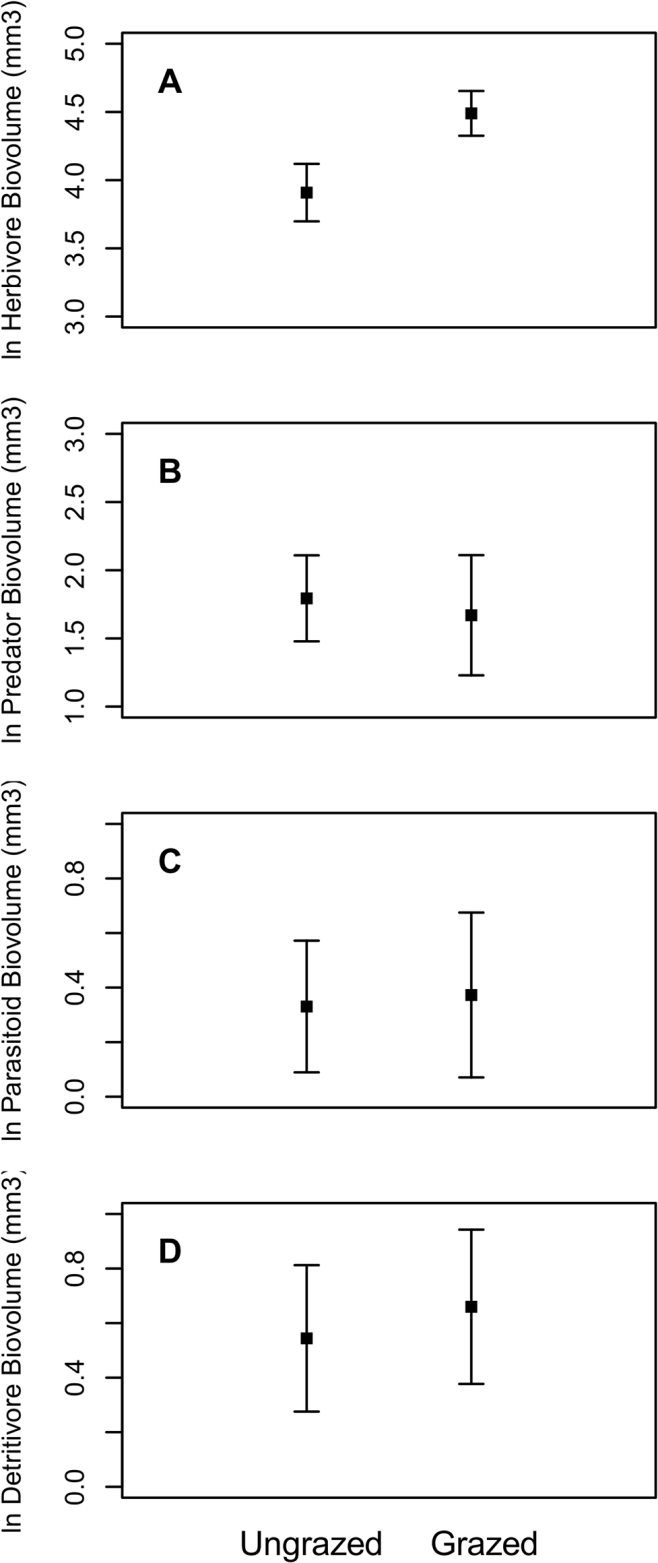
Log response of each trophic group’s biovolume to grazing treatments. Values represent means ±SE.

Because plot-scale biovolume incorporates both taxon size and abundance, we examined the effects of the experimental treatments on mean taxon size and total arthropod abundance. We found that arthropod abundance did not vary as a function of the treatments (p>0.05), but the mean biovolume of arthropod taxa in grazed annual plots was larger than the other treatments (p = 0.03). The separate biovolumes of herbivores, predators, parasites, and detritivores did not vary consistently with grass treatment (Table 2). However, herbivorous arthropod biovolume increased and predatory arthropod volume decreased with grazing after accounting for experimental block and plot vegetation characteristics (plant richness, litter mass, etc.) (F-test herbivores p = 0.0020, predators p = 0.0208; Table 2), primarily driven by biovolume changes in the grazed annual plots. The total biovolume of herbivorous arthropods was 79% higher in grazed plots, whereas the biovolume of predatory arthropods was 13% higher in ungrazed plots (Fig 3A and 3B). Neither parasitoid nor detritivore biovolume varied with grass provenance or grazing treatments (Fig 3C and 3D).

**Fig 4:**
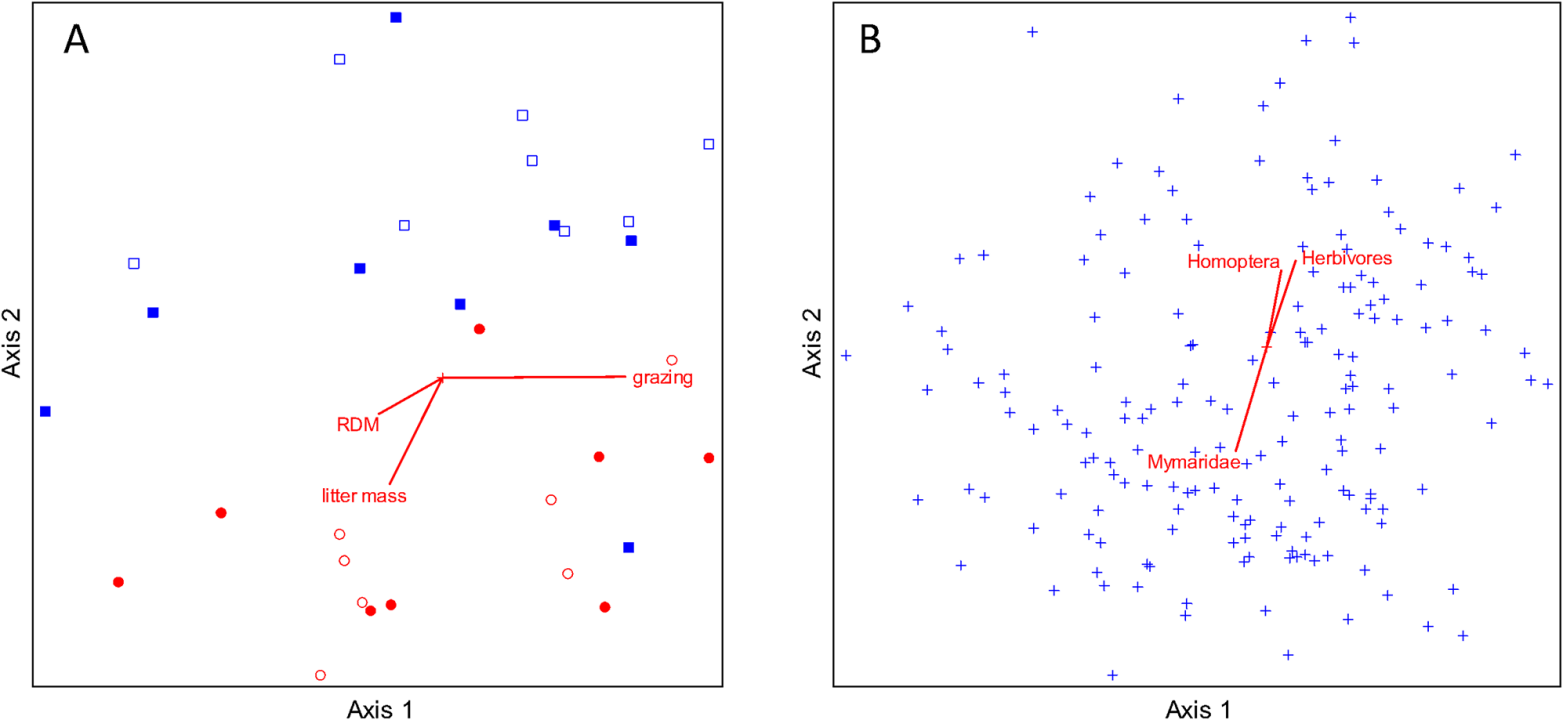
Two views of the same 4-dimentional NMS ordination of plots in arthropod species space. (A) Open shapes represent native perennial grass plots; filled shapes represent non-native annual plots. Plots in experimental field 5 are represented by blue squares; field 14 by red circles. Joint plot lines show environmental variables that were strongly correlated with the arthropod species composition as represented in the ordination. Grazing was negatively associated with litter mass and residual dry matter (RDM), and was a strong predictor of arthropod assemblage. Arthropod assemblages from the two grass treatments were not distinct from each other. (B) The same ordination, but each point represents the placement of an arthropod morphospecies within the species space. Joint plot lines show groups of arthropods whose biomass in a plot was strongly correlated with the ordination.

Grazing significantly altered the composition of the arthropod community by differentially affecting the relative abundances of morphospecies in each plot (perMANOVA F = 2.47, df = 1, p<0.001, Table 3); grazed plots had community compositions more similar to each other than to those of the ungrazed plots (Fig 4). Arthropod communities that were positively correlated with grazing were also negatively correlated with plant litter. Herbivorous arthropods, including Auchenorryncha and Sternorryncha, were more prevalent in plots containing little plant litter (Fig 4). In spite of the strong effect of grazing on arthropod composition, there was no indication that arthropod morphospecies responded to grazing as phylogenetically related groups (p = 0.40); thus, even closely related morphospecies responded independently with respect to the experimental treatments. In contrast, the proportional representation of arthropod morphospecies was the same across native and non-native grass plots in both grazed and ungrazed treatments (perMANOVA p>0.1 for all, Table 3).

**Table 3:** Results of perMANOVAs evaluating differences in proportional morphospecies abundances between experimental treatments.

perMANOVA test	# plots	# morphos	Blocking F-ratio (df)	Treatment F-ratio (df)
Grazing	30	177	1.4150**(14)	2.4749**(1)
Grass in Ungrazed	16	138	1.7347 (1)	1.2273 (2)
Grass in Grazed	12	112	1.6921**(1)	0.9557 (2)

For each test, the number of plots and the number of morphospecies used in the analysis are included. The F-ratios, their significance levels (** p< 0.001), and the degrees for freedom (df) are given for each test. Paired grazing plots were evaluated with Field as a blocking factor to account for differences in baseline morphospecies’ abundances between fields. Grass treatment was evaluated separately for grazed and ungrazed plots due to the complexity of the experimental design and were blocked by treatment block.

## Discussion

Grazing altered arthropod community composition; the effect of grazing on arthropod communities occurred regardless of vegetation characteristics. Grazing resulted in increased biomass of herbivorous arthropods and decreased biomass of predatory arthropods. These results suggest that grazing may alter plant biomass and composition directly via vegetation removal and indirectly via an altered arthropod consumer community. In contrast to our expectations, arthropod community composition was surprisingly invariant between plant communities dominated by native perennial grasses and non-native annual grasses in the absence of grazing. Thus, in spite of the substantial differences between native and non-native grasses, these grass communities support a similar arthropod community regardless of provenance in this ecosystem.

Our grazing treatments also resulted in an overall shift in arthropod community composition. This pattern may be the result of a trophic cascade, or from herbivorous and predatory arthropods responding to changes in different characteristics of the plant community. Our cattle grazing treatment reduced plant litter and RDM and caused a reduction in plant standing biomass. Predatory arthropod species composition and abundance is highly correlated with vegetation structure [16,17,34], with greater predator abundance and diversity associated with the taller, more complex vegetation structure of ungrazed habitats [18]. A reduction in predators could have led to an increase in herbivorous arthropod biomass. In contrast, herbivores could have responded to increased plant nutritional quality or productivity, which often results from grazing or cutting [15,19]. The total arthropod increase associated with grazing could also be, in part, due to decreased plant biomass in grazed plots resulting in a higher sampling rate per unit plant surface; however, this sampling effect is not likely to underlie the observed change in the ratio of herbivores to predators as arthropods from both trophic groups would be sampled at a higher rate. It is interesting to note that the compositional changes resulting from grazing, namely increased abundances of Auchenorryncha and Sternorryncha, were also associated with the lowest levels of plant litter. This is consistent with the expectation that these groups are key to ecosystem-level decomposition and nutrient recycling rates [4–6,35,36], suggesting that vertebrate grazing may stimulate more rapid ecosystem processes, in part via altered arthropod composition. Grazing may thus have a disproportionately large impact on grassland plant communities through direct and indirect means mediated via the arthropod community: it may decrease plant biomass through direct removal of plant material, by increasing herbivorous arthropod biomass, and by decreasing predator biomass, as predator absence can have large negative impacts on producer biomass across productivity gradients and habitats [37].

When grazers were present, the dominance of non-native annual grasses also led to increased total arthropod biovolume. Biovolume is the product of the size and abundance of taxa in a plot, and in this case, our results suggest turnover to larger body sizes in grazed plots with annual grasses. The non-significant effects of treatments on feeding groups suggest that this change in body size is independent of, or only weakly associated with, turnover in arthropod functional groups. Although ours is the first study to document the interactive effects of grazing and domination of non-native annual grasses on the total plot-scale mass of arthropods, our results extend earlier work demonstrating turnover of arthropod community composition within taxonomic groups in response to these joint factors [38].

Arthropod richness, evenness, abundance, and the proportional representation of arthropod morphospecies were surprisingly similar between plant communities dominated by native perennial grasses and non-native annual grasses, in spite of the substantial differences (e.g. physical structure, palatability, etc.) between these two groups of grasses. The lack of observed difference between grass treatments is not likely a sampling artifact: the substantial arthropod community responses to the grazing treatments indicate that the spatial scale of this experiment was appropriate for measuring changes in arthropod community. Prior work contrasts with these findings by demonstrating that arthropod richness can be reduced or altered in the presence of a non-native plant [23,39]; however, unlike earlier work, we found no appreciable difference in plant diversity between our native and non-native grass assemblages. Thus, this difference between our study and previous work suggests that the most important factor controlling arthropod richness in these previous studies was likely due to changes in plant diversity, rather than presence of a non-native plant. This inference is also consistent with recent work on plant and arthropod diversity relationships [14]. Further, while the grass species of our two treatments differ in life history, all species are representatives of the C3 grass functional group. Where coexistence of native and non-native plant species occurs without drastically shifting the species richness or functional composition of the plant community, our findings suggest that there may be less impact on the herbivore assemblage than when the plant invader is functionally or phylogenetically distinct or induces local declines in plant diversity. Our results suggest that grassland species composition may be less important than factors controlling total plant richness or productivity, for example, for predicting arthropod diversity [40].

A high proportion of exotic grasses in California are annuals, including all the species used in this experiment, while the majority of native grasses are perennial [20,41]; thus, grass provenance and life history are confounded in this ecosystem and in the experimental treatments. Arthropods may have higher biovolume in our non-native grazed plots because annual plants generally have less investment in structural defenses and a lower C:N ratio than their perennial counterparts [42,43]. Aphids are known to have higher fecundity on annual than perennial grasses [24], and other arthropod groups may respond similarly. In addition, grazing can stimulate annual grass productivity [19], potentially leading to significantly increased secondary production of arthropods [14]. Thus, secondary production of arthropods may be constrained by the efficiency with which these consumers access and convert primary production rather than by plant provenance, *per se* [44]. An increase in arthropod biovolume in grazed annual grasslands in California, even without altering the consumer community composition, could have substantial effects on ecosystem functions such as decomposition or nutrient recycling rates [5].

Since the world’s grasslands are expected to experience increased grazing intensity to meet heightened food demands [45] and as the ranges of exotic plant species are predicted to increase as they overcome dispersal limitation [41], the quantitative changes in arthropod community from grazing and non-native annual grasses could lead to widespread alterations of ecosystem functioning. Increased herbivorous arthropod biomass with grazing could increase grassland nutrient cycling rates [5] or increase ecosystem productivity [46]. This interaction also could initiate a positive feedback cycle in vector transmitted plant disease; annual grasses cause high fecundity in arthropod disease vectors [24,47], increasing the prevalence of plant diseases that shift competitive dominance from native perennials to exotic annuals [28], further increasing domination of the plant community by annual plants [27].

Grasslands worldwide are subject to the combined impacts of grazing and invasive species [48], and because of the key role arthropods play in community structure and function, the indirect effects of human land use alteration on arthropod communities may have important consequences for multiple ecosystem functions. For example, carbon sequestration by grasslands is an important environmental service [45] that can be decreased by vertebrate grazing and associated increased herbivorous arthropod biovolume. Ecosystem services mediated by qualitative changes in the arthropod community, however, may remain intact despite the dominance of non-native plants, as neither arthropod richness nor arthropod community structure was altered by this disturbance. The far-reaching effects of human-induced disturbances, including alterations of grassland arthropod composition and function, will likely become more pronounced as these disturbances become stronger and more widespread.

## Supporting Information

S1 Dataset Arthropod and plant data from Sierra Foothills grass provenance × grazing experiment. File is a workbook with four spreadsheets: “ArthropodCounts” contains the raw arthropod data collected in this experiment; “ArthropodMetadata” describes that spreadsheet; “PlantData” contains the plant community metrics from each experimental plot used in this analysis; and “PlantExplanation” clarifies the plant data measurements.
(XLSX)

S1 Table Table of arthropod morphospecies from Sierra Foothills grass provenance × grazing experiment, with grazing correlations. Arthropod taxa were identified by Kelly A. Farrell using keys in references [49–57]; other arthropods were identified with assistance from David Maddison, Samantha Colby, Kojun Kanda, Danielle Lightle, and crowd-sourced using the identification resource BugGuide.net. References used to determine trophic grouping are indicated with subscripts: [49–57] refer to references in manuscript, with other trophic groupings identified by the individuals referenced above. “*” preceding a trophic classification indicates that a larval foodsource is used though adults were collected.
(DOCX)
