# Supplementary material for: Grassland Arthropods Are Controlled by Direct and Indirect Interactions with Cattle but Are Largely Unaffected by Plant Provenance
Source: PLoS One. 2015 Jul 9;10(7):e0129823. doi: 10.1371/journal.pone.0129823 (PMC4497643; doi:10.1371/journal.pone.0129823)
Supplement: S1 Table — Arthropod taxa were identified by Kelly A. Farrell using keys in references [49–57]; other arthropods were identified with assistance from David Maddison, Samantha Colby, Kojun Kanda, Danielle Lightle, and crowd-sourced using the identification resource BugGuide.net. References used to determine trophic grouping are indicated with subscripts: [49–57] refer to references in manuscript, with other trophic groupings identified by the individuals referenced above. “*” preceding a trophic classification indicates that a larval foodsource is used though adults were collected. (DOCX) [file pone.0129823.s002.docx]

| Morphospecies | Order | Taxon | Trophic Grouping | Correlation with Grazing | Average Biovolume (mm^3^) | Number of Individuals | Plots with Occurrence |
| --- | --- | --- | --- | --- | --- | --- | --- |
| 104 | Acarina | Ceratozetidae_56_ |  | -0.1768 | 0.0188 | 1 | 1 |
| 903 | Acarina |  |  | -0.2404 | 0.1117 | 3 | 2 |
| 906 | Acarina |  |  | -0.1740 | 0.0061 | 139 | 3 |
| 911 | Acarina | Mesostigmata | predator | 0.2613 | 0.0393 | 14 | 5 |
| 904 | Acarina | Prostigmata | predator | -0.3169 | 0.2068 | 3 | 3 |
| 907 | Acarina | Prostigmata | predator | -0.2977 | 0.0503 | 208 | 11 |
| 908 | Acarina | Prostigmata | predator | 0.2208 | 0.1478 | 8 | 4 |
| 910 | Acarina | Prostigmata | predator | -0.0738 | 0.0063 | 17 | 8 |
| 900 | Acarina | Trombidiidae | predator | -0.2622 | 0.6413 | 68 | 10 |
| 901 | Acarina | Trombidiidae | predator | 0.4543 | 0.3058 | 615 | 28 |
| 902 | Acarina | Trombidiidae | predator | -0.2497 | 0.0767 | 18 | 9 |
| 905 | Acarina | Trombidiidae | predator | -0.0246 | 0.5737 | 42 | 11 |
| 912 | Acarina | Trombidiidae | predator | -0.1092 | 0.1676 | 6 | 4 |
| 909 | Acarina | Trombidiform | predator | 0.1886 | 0.0251 | 1 | 1 |
| 800 | Araneae |  | predator | -0.2616 | 0.6362 | 6 | 5 |
| 801 | Araneae |  | predato | -0.0616 | 0.9385 | 32 | 14 |
| 802 | Araneae |  | Predator | -0.1284 | 1.2704 | 9 | 6 |
| 803 | Araneae |  | predator_57_ | 0.3485 | 1.1221 | 150 | 24 |
| 804 | Araneae |  | predator_57_ | -0.2543 | 43.8748 | 2 | 2 |
| 805 | Araneae |  | predator_57_ | -0.1188 | 0.2797 | 71 | 17 |
| 806 | Araneae |  | predator_57_ | -0.3633 | 0.6734 | 15 | 11 |
| 807 | Araneae |  | predator_57_ | -0.3494 | 0.5821 | 5 | 4 |
| 808 | Araneae |  | predator_57_ | 0.1019 | 62.1910 | 5 | 5 |
| 809 | Araneae |  | predator_57_ | -0.2259 | 0.3011 | 4 | 2 |
| 810 | Araneae |  | predator_57_ | -0.1326 | 0.1747 | 96 | 13 |
| 811 | Araneae |  | predator_57_ | -0.1768 | 2.5133 | 1 | 1 |
| 812 | Araneae |  | predator_57_ | 0.1886 | 19.7920 | 1 | 1 |
| 813 | Araneae |  | predator_57_ | 0.2712 | 2.9845 | 2 | 2 |
| 814 | Araneae |  | predator_57_ | 0.1197 | 3.8964 | 3 | 3 |
| 956 | Collembola |  | detritivore_55_ | 0.1197 | 0.0265 | 3 | 3 |
| 1 | Collembola | Sminthuridae | detritivore_55_ | 0.1915 | 1.7230 | 14005 | 31 |
| 2 | Collembola | Sminthuridae | detritivore_55_ | -0.1768 | 0.4712 | 3 | 1 |
| 958 |  |  |  | -0.1806 | 0.0654 | 218 | 14 |
| 965 |  |  |  | -0.1768 | 1.6965 | 1 | 1 |
| 10 | Orthoptera | Acrididae | Herbivore_56_ | 0.2465 | 39.2553 | 40 | 20 |
| 32 | Thysanoptera | Thripidae | herbivore_55_ | 0.2320 | 0.4119 | 492 | 30 |
| 33 | Thysanoptera |  |  | -0.3636 | 0.0515 | 34 | 12 |
| 950 | Thysanoptera |  |  | -0.4343 | 0.2851 | 143 | 14 |
| 951 | Thysanoptera |  |  | -0.3235 | 0.1251 | 817 | 28 |
| 953 | Thysanoptera |  |  | 0.2069 | 0.6671 | 12 | 3 |
| 30 | Thysanoptera | Aeolothripidae |  | -0.0649 | 1.3404 | 220 | 27 |
| 31 | Thysanoptera |  |  | -0.2421 | 3.5111 | 767 | 31 |
| 703 | Hemiptera |  |  | 0.2535 | 11.7286 | 48 | 9 |
| 711 | Hemiptera |  |  | -0.1782 | 3.0240 | 5 | 3 |
| 733 | Hemiptera |  |  | 0.1197 | 1.2812 | 3 | 3 |
| 726 | Hemiptera |  |  | 0.1886 | 0.2011 | 1 | 1 |
| 734 | Hemiptera |  |  | 0.1886 | 3.3175 | 2 | 1 |
| 742 | Hemiptera |  |  | 0.2340 | 0.1402 | 12 | 10 |
| 743 | Hemiptera |  |  | 0.1886 | 168.5327 | 2 | 1 |
| 744 | Hemiptera |  |  | 0.1886 | 2.8510 | 1 | 1 |
| 723 | Hemiptera | Anthocoridae |  | 0.0093 | 3.2427 | 10 | 3 |
| 724 | Hemiptera | Nabidae | predator_49_ | -0.2543 | 25.0603 | 2 | 2 |
| 718 | Hemiptera | Reduviidae | predator_49_ | 0.2727 | 0.9163 | 29 | 13 |
| 722 | Hemiptera | Reduviidae | predator_49_ | 0.1886 | 63.9832 | 1 | 1 |
| 729 | Hemiptera | Nabidae | predator_49_ | 0.1886 | 68.7632 | 1 | 1 |
| 725 | Hemiptera | Lygaeidae | herbivore_49_ | 0.2712 | 2.5236 | 2 | 2 |
| 727 | Hemiptera | Lygaeoidea | herbivore_49_ | 0.0809 | 20.7325 | 7 | 6 |
| 700 | Hemiptera | Berytidae | herbivore_49_ | 0.2517 | 1.1573 | 56 | 13 |
| 707 | Hemiptera | Berytidae | herbivore_49_ | 0.0227 | 4.9943 | 12 | 11 |
| 716 | Hemiptera | Berytidae | herbivore_49_ | -0.0600 | 0.0715 | 12 | 7 |
| 719 | Hemiptera | Berytidae | herbivore_49_ | 0.3106 | 0.6160 | 6 | 3 |
| 732 | Hemiptera | Berytidae | herbivore_49_ | 0.2959 | 0.6715 | 20 | 6 |
| 728 | Hemiptera | Acanthosomatidae |  | -0.2543 | 42.2733 | 2 | 2 |
| 702 | Hemiptera | Pentatomidae |  | -0.2600 | 8.7891 | 11 | 7 |
| 704 | Hemiptera | Pentatomidae |  | -0.2854 | 49.7969 | 10 | 7 |
| 717 | Hemiptera | Pentatomidae |  | 0.3975 | 393.4428 | 4 | 4 |
| 720 | Hemiptera | Pentatomidae |  | 0.0891 | 43.9791 | 16 | 9 |
| 731 | Hemiptera | Pentatomidae |  | -0.2404 | 571.8201 | 3 | 2 |
| 736 | Hemiptera | Pentatomidae |  | 0.1985 | 96.2042 | 5 | 3 |
| 710 | Hemiptera | Pentatomidae |  | 0.2370 | 32.6260 | 22 | 6 |
| 701 | Hemiptera | Miridae |  | 0.2127 | 7.7971 | 19 | 12 |
| 705 | Hemiptera | Miridae |  | 0.0658 | 37.8928 | 54 | 18 |
| 708 | Hemiptera | Miridae |  | -0.0737 | 10.5371 | 22 | 10 |
| 712 | Hemiptera | Miridae |  | -0.0249 | 20.2077 | 13 | 9 |
| 713 | Hemiptera | Miridae |  | -0.4640 | 17.0846 | 22 | 15 |
| 714 | Hemiptera | Miridae |  | -0.1576 | 18.2417 | 11 | 10 |
| 715 | Hemiptera | Miridae |  | 0.3324 | 25.7360 | 8 | 3 |
| 721 | Hemiptera | Miridae |  | 0.2305 | 9.1106 | 6 | 2 |
| 739 | Hemiptera | Miridae |  | -0.1768 | 17.5207 | 1 | 1 |
| 741 | Hemiptera | Miridae |  | 0.1886 | 2.5447 | 1 | 1 |
| 709 | Hemiptera | Miridae |  | -0.2543 | 6.9796 | 2 | 2 |
| 960 | Hemiptera | Sternorryncha | herbivore_55_ | 0.1886 | 0.1555 | 1 | 1 |
| 973 | Hemiptera | Sternorryncha | herbivore_55_ | -0.1768 | 0.1131 | 1 | 1 |
| 605 | Hemiptera | Aleyrodidae | *herbivore_55_ | 0.2814 | 0.0731 | 35 | 12 |
| 609 | Hemiptera | Psyllidae | herbivore_55_ | -0.2922 | 0.6766 | 9 | 8 |
| 611 | Hemiptera | Psyllidae | herbivore_55_ | -0.1768 | 26.4648 | 1 | 1 |
| 600 | Hemiptera | Aphididae | herbivore_55_ | 0.2955 | 0.5266 | 982 | 31 |
| 601 | Hemiptera | Aphididae | herbivore_55_ | -0.0300 | 0.5564 | 79 | 15 |
| 603 | Hemiptera | Aphididae | herbivore_55_ | 0.2033 | 0.7474 | 34 | 17 |
| 610 | Hemiptera | Coccoidea | herbivore_55_ | -0.4252 | 0.2249 | 36 | 12 |
| 955 | Hemiptera | Coccoidea | herbivore_55_ | -0.2290 | 0.0194 | 34 | 15 |
| 969 | Hemiptera | Coccoidea | herbivore_55_ | 0.0085 | 1.3085 | 2 | 2 |
| 521 | Hemiptera | Cercopidae | herbivore_55_ | -0.2543 | 4.3982 | 2 | 2 |
| 531 | Hemiptera | Cercopidae | herbivore_55_ | 0.1886 | 2.7709 | 2 | 1 |
| 524 | Hemiptera | Delphacidae | herbivore_55_ | 0.1634 | 3.3770 | 4 | 3 |
| 500 | Hemiptera | Membracidae | herbivore_55_ | -0.1443 | 122.2576 | 18 | 9 |
| 519 | Hemiptera | Membracidae | herbivore_55_ | 0.1476 | 179.8387 | 7 | 3 |
| 502 | Hemiptera | Cicadellidae | herbivore_55_ | 0.4921 | 13.4688 | 122 | 22 |
| 503 | Hemiptera | Cicadellidae | herbivore_55_ | 0.0899 | 2.9836 | 46 | 14 |
| 504 | Hemiptera | Cicadellidae | herbivore_55_ | -0.1527 | 0.1636 | 47 | 19 |
| 505 | Hemiptera | Cicadellidae | herbivore_55_ | 0.0124 | 2.8710 | 4 | 4 |
| 506 | Hemiptera | Cicadellidae | herbivore_55_ | -0.0527 | 7.8172 | 5 | 3 |
| 507 | Hemiptera | Cicadellidae | herbivore_55_ | 0.2828 | 6.4191 | 41 | 11 |
| 508 | Hemiptera | Cicadellidae | herbivore_55_ | 0.2266 | 0.3048 | 103 | 20 |
| 509 | Hemiptera | Cicadellidae | herbivore_55_ | -0.0986 | 1.7230 | 3 | 3 |
| 510 | Hemiptera | Cicadellidae | herbivore_55_ | -0.1235 | 11.7747 | 59 | 24 |
| 511 | Hemiptera | Cicadellidae | herbivore_55_ | -0.1719 | 11.1614 | 10 | 7 |
| 512 | Hemiptera | Cicadellidae | herbivore_55_ | 0.4981 | 2.5133 | 29 | 16 |
| 513 | Hemiptera | Cicadellidae | herbivore_55_ | 0.1886 | 0.5105 | 1 | 1 |
| 514 | Hemiptera | Cicadellidae | herbivore_55_ | 0.2712 | 0.5890 | 2 | 2 |
| 515 | Hemiptera | Cicadellidae | herbivore_55_ | 0.1363 | 0.5342 | 99 | 19 |
| 516 | Hemiptera | Cicadellidae | herbivore_55_ | 0.1886 | 0.0440 | 1 | 1 |
| 517 | Hemiptera | Cicadellidae | herbivore_55_ | 0.1357 | 0.2464 | 16 | 8 |
| 518 | Hemiptera | Cicadellidae | herbivore_55_ | 0.1634 | 2.2774 | 4 | 3 |
| 520 | Hemiptera | Cicadellidae | herbivore_55_ | 0.1197 | 3.6128 | 3 | 3 |
| 522 | Hemiptera | Cicadellidae | herbivore_55_ | -0.2543 | 2.5635 | 2 | 2 |
| 523 | Hemiptera | Cicadellidae | herbivore_55_ | 0.0719 | 2.9103 | 9 | 7 |
| 525 | Hemiptera | Cicadellidae | herbivore_55_ | -0.1801 | 29.1912 | 4 | 4 |
| 526 | Hemiptera | Cicadellidae | herbivore_55_ | 0.1886 | 12.3150 | 1 | 1 |
| 527 | Hemiptera | Cicadellidae | herbivore_55_ | 0.1526 | 4.7393 | 39 | 7 |
| 528 | Hemiptera | Cicadellidae | herbivore_55_ | -0.0986 | 6.5531 | 3 | 3 |
| 529 | Hemiptera | Cicadellidae | herbivore_55_ | 0.2894 | 6.7544 | 21 | 7 |
| 530 | Hemiptera | Cicadellidae | herbivore_55_ | 0.1886 | 21.5592 | 1 | 1 |
| 501 | Hemiptera | Cicadellidae | herbivore_55_ | 0.6069 | 3.1881 | 193 | 21 |
| 533 | Hemiptera | Cicadellidae | herbivore_55_ | 0.1886 | 0.6786 | 1 | 1 |
| 106 | Coleoptera | Chrysomelidae: Bruchinae | *herbivore_52_ | 0.2564 | 4.0901 | 3 | 2 |
| 101 | Coleoptera | Lathridiidae | fungivore_52_ | 0.3570 | 0.6362 | 14 | 8 |
| 107 | Coleoptera | Lathridiidae | fungivore_52_ | 0.1886 | 2.1901 | 2 | 1 |
| 109 | Coleoptera | Anobiidae |  | -0.1768 | 5.2025 | 1 | 1 |
| 103 | Coleoptera | Corylophidae | fungivore_52_ | 0.1886 | 0.1963 | 1 | 1 |
| 111 | Coleoptera |  |  | 0.1886 | 5.5748 | 1 | 1 |
| 954 | Coleoptera_11_ |  |  | -0.1768 | 0.3848 | 2 | 1 |
| 963 | Coleoptera_11_ |  |  | -0.1768 | 0.0377 | 1 | 1 |
| 105 | Coleoptera | Coccinellidae | predator_52_ | -0.0928 | 56.0483 | 15 | 10 |
| 110 | Coleoptera | Coccinellidae | predator_52_ | -0.1768 | 0.6220 | 1 | 1 |
| 108 | Coleoptera | Coccinellidae | predator_52_ | 0.0085 | 5.3093 | 2 | 2 |
| 100 | Coleoptera | Melyridae: Dasytinae |  | -0.0696 | 5.7674 | 12 | 8 |
| 102 | Coleoptera | Melyridae: Dasytinae |  | 0.1019 | 23.1633 | 10 | 6 |
| 959 | Neuroptera | Chrysopidae |  | 0.1886 | 7.0686 | 1 | 1 |
| 964 | Neuroptera | Hemerobiidae | predator_55_ | -0.1768 | 1.2174 | 1 | 1 |
| 200 | Lepidoptera |  | Herbivore | -0.1768 | 15.2681 | 2 | 1 |
| 962 | Lepidoptera |  | Herbivore | 0.1886 | 0.5027 | 1 | 1 |
| 966 | Lepidoptera |  | Herbivore | 0.1886 | 64.1120 | 3 | 1 |
| 972 | Lepidoptera |  | Herbivore | -0.1768 | 0.1979 | 14 | 1 |
| 305 | Diptera |  |  | 0.0085 | 0.4320 | 2 | 2 |
| 309 | Diptera |  |  | 0.4013 | 1.2017 | 22 | 13 |
| 313 | Diptera |  |  | 0.0099 | 0.2464 | 4 | 3 |
| 304.6 | Diptera |  |  | -0.1768 | 0.1131 | 1 | 1 |
| 314 | Diptera |  |  | 0.1876 | 0.3334 | 8 | 3 |
| 319 | Diptera |  |  | 0.1886 | 0.1508 | 2 | 1 |
| 324 | Diptera |  |  | -0.1768 | 0.0565 | 1 | 1 |
| 326 | Diptera |  |  | -0.1768 | 2.6012 | 2 | 1 |
| 327 | Diptera |  |  | -0.1768 | 0.5890 | 1 | 1 |
| 329 | Diptera |  |  | -0.1768 | 0.8639 | 1 | 1 |
| 332 | Diptera |  |  | -0.1768 | 0.4775 | 1 | 1 |
| 333 | Diptera |  |  | 0.1886 | 63.8136 | 1 | 1 |
| 317 | Diptera |  |  | -0.3574 | 0.1764 | 12 | 7 |
| 321 | Diptera |  |  | 0.0437 | 0.0639 | 12 | 5 |
| 307 | Diptera | Sciaridae | detritivore_53_ | 0.1272 | 0.1026 | 11 | 8 |
| 308 | Diptera | Sciaridae | detritivore_53_ | / | / | 9 | 5 |
| 311 | Diptera | Sciaridae | detritivore_53_ | -0.1212 | 0.1372 | 15 | 7 |
| 316 | Diptera | Chironomidae |  | -0.2625 | 0.4954 | 11 | 3 |
| 304 | Diptera | Ceratopogonidae | predator | 0.1197 | 0.8294 | 6 | 4 |
| 310 | Diptera | Ceratopogonidae | predator | -0.2681 | 0.4506 | 21 | 6 |
| 312 | Diptera | Ceratopogonidae: *Culicoides* | predator | -0.1514 | 0.6578 | 68 | 12 |
| 302 | Diptera | Pipunculidae | parasitoid_54_ | 0.2564 | 8.5903 | 3 | 2 |
| 300 | Diptera | Phoridae |  | 0.2045 | 0.3147 | 24 | 14 |
| 322 | Diptera | Phoridae |  | -0.2543 | 0.0798 | 2 | 2 |
| 318 | Diptera | Muscidae |  | -0.2543 | 39.5608 | 4 | 2 |
| 325 | Diptera | Tachinidae | *parasite_54_ | -0.1768 | 31.7552 | 1 | 1 |
| 306 | Diptera | Scathophagidae |  | -0.1582 | 0.3329 | 22 | 9 |
| 320 | Diptera | Chloropidae |  | 0.3381 | 4.4441 | 3 | 3 |
| 301 | Diptera | Heleomyzidae: *Trixoscelis* | *detritivore_55_ | 0.0531 | 14.6641 | 65 | 13 |
| 315 | Diptera | Tephrididae |  | -0.0986 | 12.1857 | 3 | 3 |
| 323 | Diptera | Simuliidae | predator_53_ | 0.0085 | 0.3519 | 2 | 2 |
| 499 | Hymenoptera | Formicidae |  | / | / | 2 | 2 |
| 427 | Hymenoptera | Chrisidoidea | parasitoid_50_ | -0.0986 | 0.8508 | 3 | 3 |
| 303 | Hymenoptera |  |  | 0.0085 | 2.2117 | 2 | 2 |
| 429 | Hymenoptera |  | parasitoid_53_ | 0.4391 | 0.1198 | 31 | 15 |
| 433 | Hymenoptera |  | parasitoid_53_ | -0.2543 | 0.2686 | 2 | 2 |
| 442 | Hymenoptera |  | parasitoid_50_ | 0.1886 | 0.0079 | 1 | 1 |
| 467 | Hymenoptera |  | parasitoid_50_ | -0.1768 | 0.0628 | 1 | 1 |
| 4xy | Hymenoptera |  | parasitoid_50_ | / | / | 1 | 1 |
| 961 | Hymenoptera |  |  | -0.1768 | 0.0377 | 1 | 1 |
| 414 | Hymenoptera | Ceraphronidae | parasitoid_50_ | 0.1634 | 0.1696 | 4 | 3 |
| 462 | Hymenoptera | Diapriidae: *Ambositrinae* | parasitoid_50_ | 0.0085 | 0.1272 | 2 | 2 |
| 454 | Hymenoptera | Cynipoidea | herbivore_50_ | 0.1886 | 1.3085 | 1 | 1 |
| 458 | Hymenoptera | Platygastroidea | parasitoid_50_ | 0.0085 | 0.0503 | 2 | 2 |
| 416 | Hymenoptera | Platygastridae | parasitoid_50_ | 0.1151 | 0.0524 | 9 | 5 |
| 464 | Hymenoptera | Platygastridae | parasitoid_50_ | 0.1886 | 0.0173 | 2 | 1 |
| 445 | Hymenoptera | Ichneumonoi-dea | parasitoid_50_ | -0.1436 | 0.5815 | 4 | 3 |
| 404 | Hymenoptera | Ichneumonidae: *Phygadeuontini* | parasitoid_50_ | 0.2712 | 0.4273 | 2 | 2 |
| 405 | Hymenoptera | Brachionidae | parasitoid_50_ | -0.1475 | 0.3829 | 7 | 5 |
| 411 | Hymenoptera | Brachionidae | parasitoid_50_ | -0.2543 | 1.4137 | 2 | 2 |
| 446 | Hymenoptera | Brachionidae | parasitoid_50_ | -0.1768 | 0.0503 | 1 | 1 |
| 456 | Hymenoptera | Brachionidae | parasitoid_50_ | 0.1886 | 13.1193 | 1 | 1 |
| 459 | Hymenoptera | Brachionidae | parasitoid_50_ | 0.1886 | 0.0565 | 1 | 1 |
| 469 | Hymenoptera | Vanhoriidae | parasitoid_50_ | 0.1886 | 2.7709 | 1 | 1 |
| 4xx | Hymenoptera | Chalcidoidea | parasitoid_50_ | / | / | 1 | 1 |
| 400 | Hymenoptera | Chalcidoidea | parasitoid_50_ | 0.0085 | 0.1979 | 2 | 2 |
| 401 | Hymenoptera | Chalcidoidea | parasitoid_50_ | -0.1436 | 1.8064 | 4 | 3 |
| 402 | Hymenoptera | Chalcidoidea | parasitoid_50_ | -0.3116 | 0.9702 | 8 | 3 |
| 406 | Hymenoptera | Chalcidoidea | parasitoid_50_ | / | / | 1 | 1 |
| 406.3 | Hymenoptera | Chalcidoidea | parasitoid_50_ | -0.1768 | 0.6676 | 1 | 1 |
| 407 | Hymenoptera | Chalcidoidea | parasitoid_50_ | 0.2201 | 0.2575 | 32 | 20 |
| 408 | Hymenoptera | Chalcidoidea | parasitoid_50_ | -0.1768 | 0.1131 | 1 | 1 |
| 417 | Hymenoptera | Chalcidoidea | parasitoid_50_ | -0.1768 | 0.0565 | 1 | 1 |
| 418 | Hymenoptera | Chalcidoidea | parasitoid_50_ | 0.2564 | 0.2545 | 3 | 2 |
| 419 | Hymenoptera | Chalcidoidea | parasitoid_50_ | -0.1909 | 0.0638 | 6 | 3 |
| 423 | Hymenoptera | Chalcidoidea | parasitoid_50_ | 0.1886 | 0.1272 | 1 | 1 |
| 424 | Hymenoptera | Chalcidoidea | parasitoid_50_ | 0.0085 | 0.0834 | 2 | 2 |
| 425 | Hymenoptera | Chalcidoidea | parasitoid_50_ | 0.3789 | 0.3598 | 39 | 11 |
| 426 | Hymenoptera | Chalcidoidea | parasitoid_50_ | -0.0993 | 0.0740 | 8 | 6 |
| 428 | Hymenoptera | Chalcidoidea | parasitoid_50_ | 0.2712 | 0.1424 | 2 | 2 |
| 437 | Hymenoptera | Chalcidoidea | parasitoid_50_ | -0.1768 | 0.1272 | 1 | 1 |
| 449 | Hymenoptera | Chalcidoidea | parasitoid_50_ | 0.2050 | 0.0914 | 4 | 4 |
| 451 | Hymenoptera | Chalcidoidea | parasitoid_50_ | -0.1768 | 0.1367 | 4 | 1 |
| 457 | Hymenoptera | Chalcidoidea | parasitoid_50_ | -0.0986 | 0.3016 | 3 | 3 |
| 460 | Hymenoptera | Chalcidoidea | parasitoid_50_ | -0.1959 | 1.1027 | 10 | 2 |
| 461 | Hymenoptera | Chalcidoidea | parasitoid_50_ | -0.1747 | 0.6414 | 11 | 3 |
| 450 | Hymenoptera | Eurytomidae: *Sycophila* | parasitoid_50_ | -0.1768 | 0.5278 | 1 | 1 |
| 465 | Hymenoptera | Chalcidoidea | parasitoid_50_ | 0.1886 | 4.2237 | 20 | 1 |
| 466 | Hymenoptera | Chalcidoidea | parasitoid_50_ | -0.1768 | 0.5781 | 2 | 1 |
| 468 | Hymenoptera | Chalcidoidea | parasitoid_50_ | 0.1886 | 0.2969 | 2 | 1 |
| 452 | Hymenoptera | Encyrtidae | parasitoid_50_ | -0.1436 | 0.5701 | 4 | 3 |
| 406.2 | Hymenoptera | Eulophidae | parasitoid_50_ | -0.0291 | 0.2118 | 43 | 17 |
| 444 | Hymenoptera | Eulophidae | parasitoid_50_ | -0.1768 | 0.1979 | 1 | 1 |
| 448 | Hymenoptera | Eulophidae | parasitoid_50_ | 0.1886 | 0.4775 | 1 | 1 |
| 415 | Hymenoptera | Eupelmidae | parasitoid_50_ | -0.1049 | 0.9480 | 13 | 8 |
| 440 | Hymenoptera | Pteromalidae | parasitoid_50_ | -0.2970 | 0.7180 | 4 | 3 |
| 439 | Hymenoptera | Torymidae | parasitoid_50_ | -0.2670 | 1.4434 | 9 | 5 |
| 412 | Hymenoptera | Torymidae | parasitoid_50_ | -0.2294 | 2.4522 | 11 | 5 |
| 453 | Hymenoptera | Torymidae | parasitoid_50_ | -0.2543 | 0.3560 | 2 | 2 |
| 421 | Hymenoptera | Trichogrammati-dae | parasitoid_50_ | -0.3758 | 0.0487 | 41 | 14 |
| 432 | Hymenoptera | Trichogrammati-dae | parasitoid_50_ | -0.0214 | 0.0083 | 54 | 16 |
| 403 | Hymenoptera | Aphelinidae | parasitoid_50_ | -0.0627 | 0.0482 | 14 | 10 |
| 409 | Hymenoptera | Aphelinidae | parasitoid_50_ | 0.3545 | 0.3534 | 7 | 4 |
| 420 | Hymenoptera | Aphelinidae | parasitoid_50_ | 0.0124 | 0.0440 | 4 | 4 |
| 410 | Hymenoptera | Mymaridae | parasitoid_50_ | 0.2128 | 0.0121 | 27 | 12 |
| 413 | Hymenoptera | Mymaridae | parasitoid_50_ | -0.3588 | 0.0236 | 15 | 8 |
| 421.2 | Hymenoptera | Mymaridae | parasitoid_50_ | 0.0809 | 0.0305 | 7 | 6 |
| 430 | Hymenoptera | Mymaridae | parasitoid_50_ | -0.0739 | 0.0356 | 8 | 4 |
| 431 | Hymenoptera | Mymaridae | parasitoid_50_ | 0.0085 | 0.0134 | 2 | 2 |
| 434 | Hymenoptera | Mymaridae | parasitoid_50_ | -0.2654 | 0.0276 | 10 | 7 |
| 435 | Hymenoptera | Mymaridae | parasitoid_50_ | -0.2543 | 0.0534 | 2 | 2 |
| 436 | Hymenoptera | Mymaridae | parasitoid_50_ | 0.2561 | 0.1108 | 11 | 4 |
| 441 | Hymenoptera | Mymaridae | parasitoid_50_ | -0.0736 | 0.0098 | 5 | 5 |
| 443 | Hymenoptera | Mymaridae | parasitoid_50_ | 0.1886 | 0.0157 | 2 | 1 |
| 447 | Hymenoptera | Mymaridae | parasitoid_50_ | -0.2404 | 0.0157 | 3 | 2 |
| 455 | Hymenoptera | Mymaridae | parasitoid_50_ | -0.1768 | 0.0503 | 1 | 1 |
|  |  |  |  |  |  |  |  |
